# A culturomics approach reveals cross-feeding capacity of intestinal pig bacteria upon release of inositol from phytate

**DOI:** 10.1186/s40168-025-02313-5

**Published:** 2026-01-21

**Authors:** Lena-Sophie Paul, Michael Weber, Stefanie Wagner, Thilo M. Fuchs

**Affiliations:** Friedrich-Loeffler-Institut, Institute of Molecular Pathogenesis, Naumburger Str. 96a, Jena, 07743 Germany

**Keywords:** Phytate, *Myo*-inositol, Phytase, Catabolic pathway, Swine microbiota, Bacterial metabolism, Genomics, Cross-feeding, Pathogens

## Abstract

**Background:**

Phytate is the primary phosphorus storage molecule of plants and plays a major role in animal nutrition. To enhance phosphate availability and absorption in livestock, and to reduce eutrophication by liquid manure, bacterial phytases are often added to animal feed. The dephosphorylated form of phytate, the polyol *myo*-inositol (*myo*-Ins) with multiple functions in eukaryotes, is metabolized by approximately 30% of all bacterial species.

**Results:**

Here, we employed a culturomics approach to identify possible metabolic interactions between phytase-producing and *myo*-Ins degrading bacteria in intestinal samples from pigs. Selective cultivation revealed an unexpectedly high abundance of *myo*-Ins degrading bacteria, suggesting substantial phytate dephosphorylation in the pig gut. Phytase activity assays performed on gut isolates showed a high degree of variability, suggesting the presence of a diverse set of phytases yet to be characterized. Furthermore, using supernatants of phytase-positive gut strains cultivated in the presence of phytate, we observed cross-feeding of *myo*-Ins from phytase producers to phytase-negative strains, including the pathogen *Salmonella enterica* serovar Typhimurium.

**Conclusions:**

The data demonstrate that a wide range of commensal bacteria can potentially benefit from phytase activity by utilizing *myo*-Ins, released through phytate hydrolysis, as a growth substrate.

Video Abstract

**Supplementary Information:**

The online version contains supplementary material available at 10.1186/s40168-025-02313-5.

## Introduction

Phytate (or phytic acid, InsP_6_) is the main storage form of phosphorus and minerals in plants and is found in plant tissues such as bran and seeds of legumes and cereals, including oil seeds and nuts. In soil organic matter, phytate is the most recalcitrant phosphorus-containing compound [1]. Phytate has both beneficial and detrimental effects on health: it forms complexes not only with dietary zinc and iron, but also with positively charged food components, possibly leading to mineral-related deficiencies, reduced digestibility of some proteins, and a weaker glucose response, i.e., a lower glycemic index [2]. However, phytate also exhibits several health-promoting properties for both humans and animals. It functions as an antioxidant of food components by chelating free iron, thereby preventing iron-driven hydroxyl radical formation [3]. Additionally, phytate positively influences the composition of cecal organic acids and mucins produced by epithelial cells [4, 5]. Therapeutic benefits have been reported for a wide spectrum of diseases, including prevention or protection against colon cancer, mammary carcinoma, pancreatic cancer, HIV, and a reduction in the risk of coronary heart disease and diabetes mellitus [2]. In pigs, dietary-derived phosphorus has been shown to affect gut microbial composition, supporting a role for the commensal microbiota in the degradation of phytate [6, 7].

The phosphate is stepwisely removed from phytate by phosphohydrolytic enzymes, the phytases [8]. Phytases are primarily of microbial or plant origin and are found in soil, aquatic environments, and in the digestive system. An overview of phytase nomenclature and function has been provided by Sommerfeld [9]. Chemically, these enzymes are defined as *myo*-inositol (*myo*-Ins) (1,2,3,4,5,6) hexakisphosphate phosphohydrolases, catalyzing the stepwise release of phosphate groups [2]. Histidine acid phytases (HAPhys), alkaline β-propeller phytases (BPPhys), and protein tyrosine phytases (PTPhys) are produced by bacteria [8], whereas the purple acid phytases (PAPhys) are specific to plants. Cysteine phytases, mainly produced by ruminal bacteria, belong to the PTPhy group [10].

Plant-derived phytases either fail to fully hydrolyse phytate in food or become inactivated during food processing [11]. As a result, inositol phosphates have been reported to account for 2%–60% of total organic phosphorus in animal waste [12]. Incompletely dephosphorylated phytate (InsP_1–6_) excreted particularly by monogastric animals is subsequently cleaved by soil and water-borne microorganisms [13], contributing to eutrophication [11] that includes the proliferation of toxin-producing cyanobacteria [14]. Reducing phosphate loads therefore requires strategies like supplementing livestock diets with phytases to enhance phytate-derived phosphate absorption [15] and to mitigate environmental pollution. Consequently, microbial phytases such as the heat-labile phytases PhyA from *Aspergillus niger* or PhyE1/2 from *Escherichia coli* are commonly added to animal feed, particularly for swine and poultry. Among these, AppA from *E. coli* exhibits the highest reported catalytic turnover number [16, 17], whereas a phytase from *Citrobacter braakii* shows the highest specific activity [18].

It is assumed that the stepwise enzymatic hydrolysis of phytate generates lower *myo*-Ins phosphate esters (IP_1_-IP_6_), ultimately leading to the production of phosphate-free *myo*-Ins [2]. Recently, phytate and *myo*-Ins were identified as substrates in metabolic interactions. The intestinal species *Mitsuokella jalaludinii* was identified as an efficient phytate degrader, supplying inositol to *Anaerostipes rhamnosivorans*, enabling the synergistic production of the short-chain fatty acid propionate [19, 20].

Since *myo*-Ins is a building block for phosphatidylinositol, its derivatives, and other membrane molecules of eukaryotes [21], this polyol circulates in the mammalian bloodstream and is also present in the gut due to epithelial cell shedding [21]. *myo*-Ins is detectable in soil [22] and serves as a readily available carbon and energy source for microorganisms [23]. To date, the ability to catabolize *myo*-Ins has been experimentally confirmed in only a limited number of bacterial species, among them *Rhizobium leguminosarum* [24], *Sinorhizobium meliloti* [25], *Lactobacillus casei* [26], *Klebsiella aerogenes* [27], *Corynebacterium glutamicum* [28], *Legionella pneumophila* [29], *Yersinia mollaretii* [30], and *Citrobacter koseri* [31]. In *Bacillus subtilis,* the transporters, the regulatory repressor, and the enzymes involved in *myo*-Ins-utilization have been thoroughly characterized [32, 33], whereas the enteropathogen *S. enterica* has been used to elucidate the complex regulatory network controlling the *myo*-Ins catabolic pathway [34–38]. In a recent kingdom-wide in silico survey, we identified genes involved in inositol degradation in approximately 6300 bacterial species, representing about 29% of all bacteria with validated genomes. Notably, around 17% of human gut species were found to possess the capacity to utilize *myo*-Ins as a carbon and energy source [23].

Both phytate and *myo*-Ins play important roles in both human and animal health and contribute significantly to the global phosphorus cycle [39]. However, major gaps remain in our understanding of how intestinal bacteria contribute to the metabolism of these molecules. In particular, the enzymatic capacities of gut commensals to (partially) dephosphorylate phytate are largely unknown. Using a culturomics approach, we characterized a non-redundant set of species isolated from piglet fecal samples. We determined the proportion of *myo*-Ins degrading bacteria, quantified phytase activity among gut commensals, and demonstrated cross-feeding interactions between commensal gut bacteria that release or utilize *myo*-Ins following phytate dephosphorylation.

## Results

### Percentage of myo-Ins-utilizing bacteria in piglet feces cultivated on LB medium

**Fig. 1:**
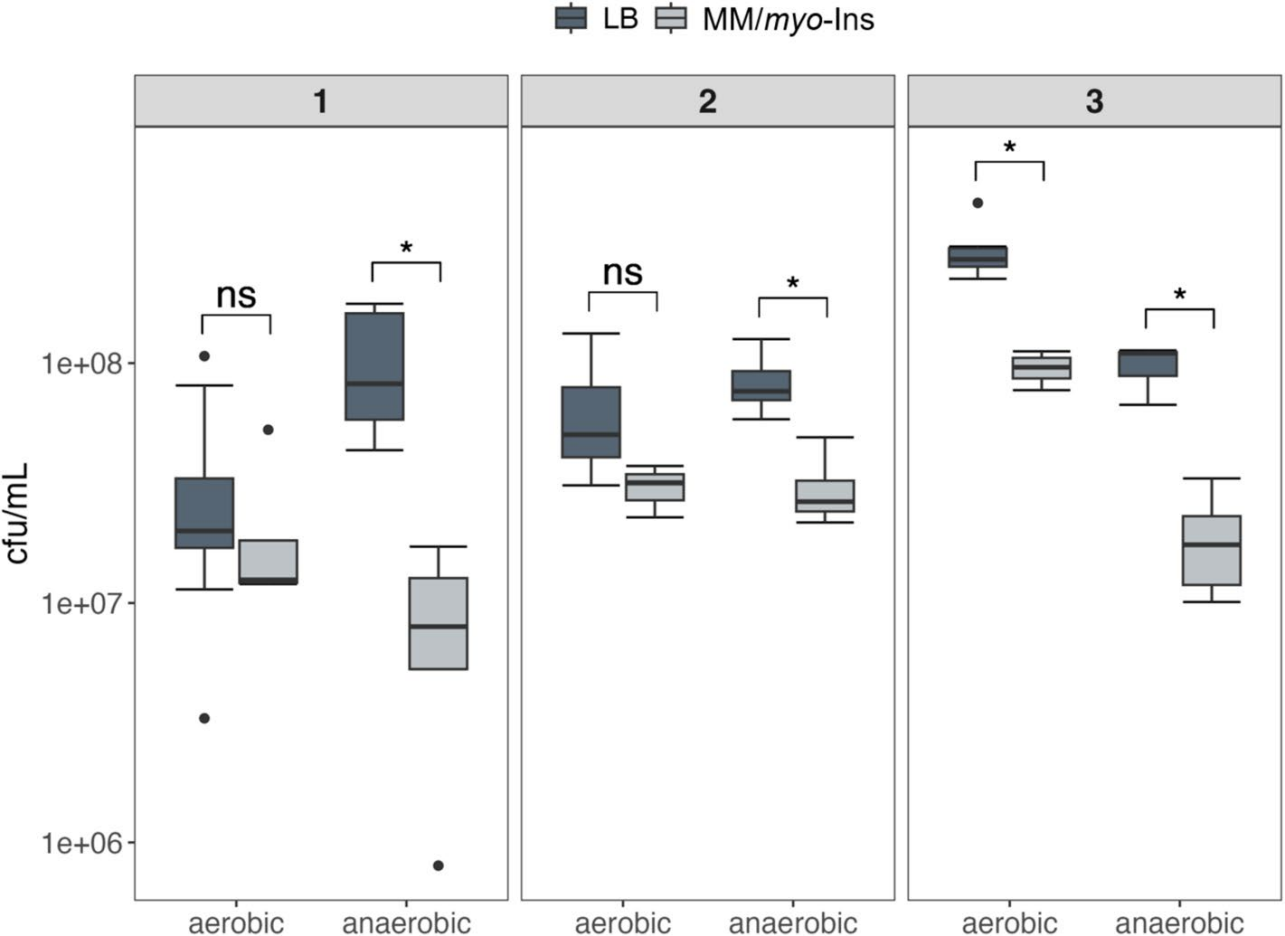
Percentage of *myo*-Ins degrading bacteria upon cultivation. Aliquots of fecal samples from three piglets were plated on LB agar and MM agar supplemented with 55 mM *myo*-Ins. Boxplots show colony forming unit counts after 24 h of incubation at 37 °C under both anaerobic and aerobic conditions. Three biologically independent replicates were performed, each with three technical replicates. p-values were determined using the Mann–Whitney nonparametric rank-sum test to compare results between media. Asterisks denote statistically significant differences (*p* < 0.05)

To determine the proportion of piglet gut bacteria capable of growing with *myo*-Ins, the final product of complete phytate dephosphorylation, we analysed fecal samples from three animals [40]. Serial dilutions of the samples were plated on agar plates containing either minimal medium (MM) without or supplemented with *myo*-Ins, or LB medium, and incubated at 37 °C under both aerobic and anaerobic conditions. Colonies were enumerated, and the proportion of *myo*-Ins-utilizing bacteria was compared to that of bacteria growing on LB medium. No bacterial growth was observed on plates lacking a carbon source. Under aerobic conditions, a higher percentage of bacterial cells were able to utilize *myo*-Ins compared to anaerobic cultivation, with values ranging from 33 to 70% across the three piglet samples. In the absence of oxygen, 17% to 39% of colonies growing on LB medium were capable of catabolizing *myo*-Ins (Fig. 1, Table S1). Moreover, we observed a binary morphological variance on *myo*-Ins agar plates, which, unlike LB plates, displayed both large and very small colonies. This observation reminds to the bistable growth behaviour among commensal bacteria for this substrate, as previously reported for *Salmonella enterica* [41, 42]. We conclude that a substantial proportion of swine gut bacteria, in particular (facultatively) aerobic cells, can grow with *myo*-Ins as their sole carbon and energy source.

### Microbiota analysis of bacteria cultivable on LB and myo-Ins medium

**Fig. 2:**
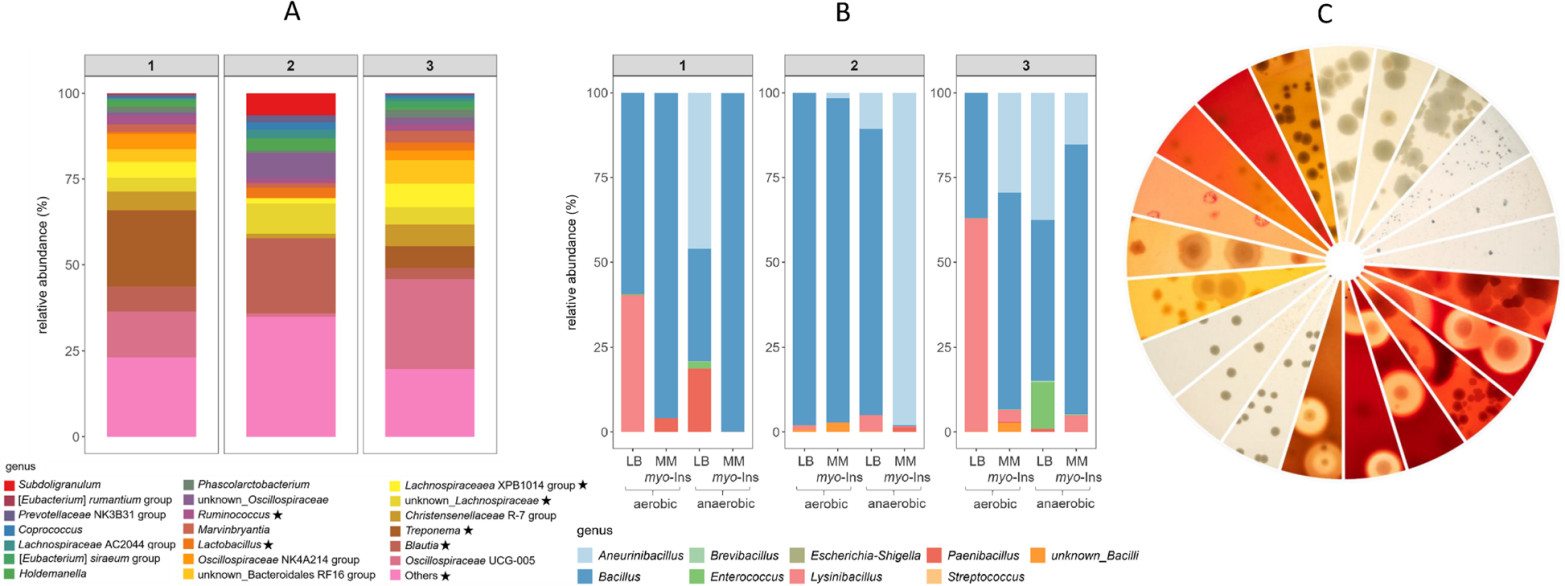
Microbiota analyses of feces samples from piglets. **A** 16S rRNA gene amplicon sequencing data derived from the samples shown in Fig. 1 were analysed. Stacked bar charts display the relative abundances of the most dominant genera. Asterisks indicate genera possessing the genetic capacity (IolCatGC) to utilize *myo*-Ins [23]. **B** Culture-dependent microbiota composition, obtained by sweeping bacteria from *myo*-Ins-containing medium. Stacked bar charts show the relative abundances of bacterial taxa. The same samples, piglets, and cultivation conditions as in **A** were used. **C** Segments from different petri dishes illustrate the isolation of distinct swine gut bacteria through culturomics

To characterize the composition and abundance of *myo*-Ins degrading bacteria in gut samples, we performed 16S rRNA gene sequence analyses of the three fecal samples described above. Determination of the relative bacterial composition revealed the predominance of 20 distinct genera, five of which include species capable of degrading *myo*-Ins, namely *Blautia*, *Lachnospiraceae* XPB1014 group, *Lactobacillus*, *Ruminococcus*, and *Treponema* (Fig. 2A) [23]. To identify the microbiota present on LB and MM/*myo*-Ins agar plates, we swept off the colonies from the plates, and the resulting suspensions were subjected to 16S rRNA gene sequencing. This culture-dependent analysis revealed in particular the presence of five genera in the fecal samples, namely *Bacillus*, *Aneurinibacillus*, *Lysinibacillus*, *Paenibacillus*, and *Enterococcus,* with relative abundances varying according to the growth medium (Fig. 2B). According to our previous large-scale in silico analysis of bacterial genomes, all five genera contain species able to degrade *myo*-Ins [23]. The clear discrepancy between culture-independent (Fig. 2A) and culture-dependent (Fig. 2B) results suggests that the growth conditions used were sub-optimal for certain bacteria, particularly anaerobes. Possible reasons include the lack of supplements, the absence of spore inhibitors, incorrect atmosphere, insufficient degassing of media, and inherent sequence limitations for species-level identification [43–45], such as clustering of *Bacillus* with *Lactobacillus*, or too low abundance of *Bacillus* in the original gut samples. These data suggest that the presence of *myo*-Ins selects for *myo*-Ins-degrading bacteria from the phylum Bacillota from the swine gut microbiota.

### Identification of gut bacteria able to catabolize myo-Ins

To establish a non-redundant collection of swine gut bacteria, we plated dilutions of fecal and gut section samples from three piglets onto a range of rich and selective culture media, which were incubated under both aerobic and anaerobic conditions (Table S2; Fig. 2C) [46–48]. However, several selective media identified none, or only a single representative of the respective genus capable to utilize *myo*-Ins [23]. In total, 195 aerobically and 190 anaerobically grown colonies were visually selected to maximize diversity in colony morphology, colour, size, and surface structure, and were subsequently isolated on appropriate media plates. Next, all 385 isolates were streaked on MM/*myo*-Ins agar, of which 175 showed growth on *myo*-Ins, a majority under aerobic conditions. For identification, the 16S rRNA genes of 149 isolates (Table S3) taken from plates with LB agar (72 isolates), Columbia blood agar (27), brain heart infusion agar (11), fastidious anaerobe agar (11), de Man-Rogosa-Sharp agar (7), Schaedler anaerobe agar (3), and MacConkey agar (18) were sequenced. Twenty-six isolates were not identified due to technical problems with DNA isolation, PCR, or sequencing. An additional 108 isolates (Table S3) were obtained from direct plating of fecal samples on MM agar containing *myo*-Ins. 16S rRNA gene sequencing, followed by SILVA- and Metaphlan-based analysis, led to the identification of 40 distinct species (Table S3). For *E. coli* and *Bacillus* sp. NBRC 100906, two isolates were listed due to differences in colony morphology and the SILVA analysis results. With the exception of four species belonging to the phylum Pseudomonadota, all non-redundant isolates were members of the phylum Bacillota and its genera *Aneurinibacillus*, *Bacillus*, *Brevibacillus*, *Lysinibacillus*, *Rummeliibacillus*, and *Paenibacillus*.

**Fig. 3:**
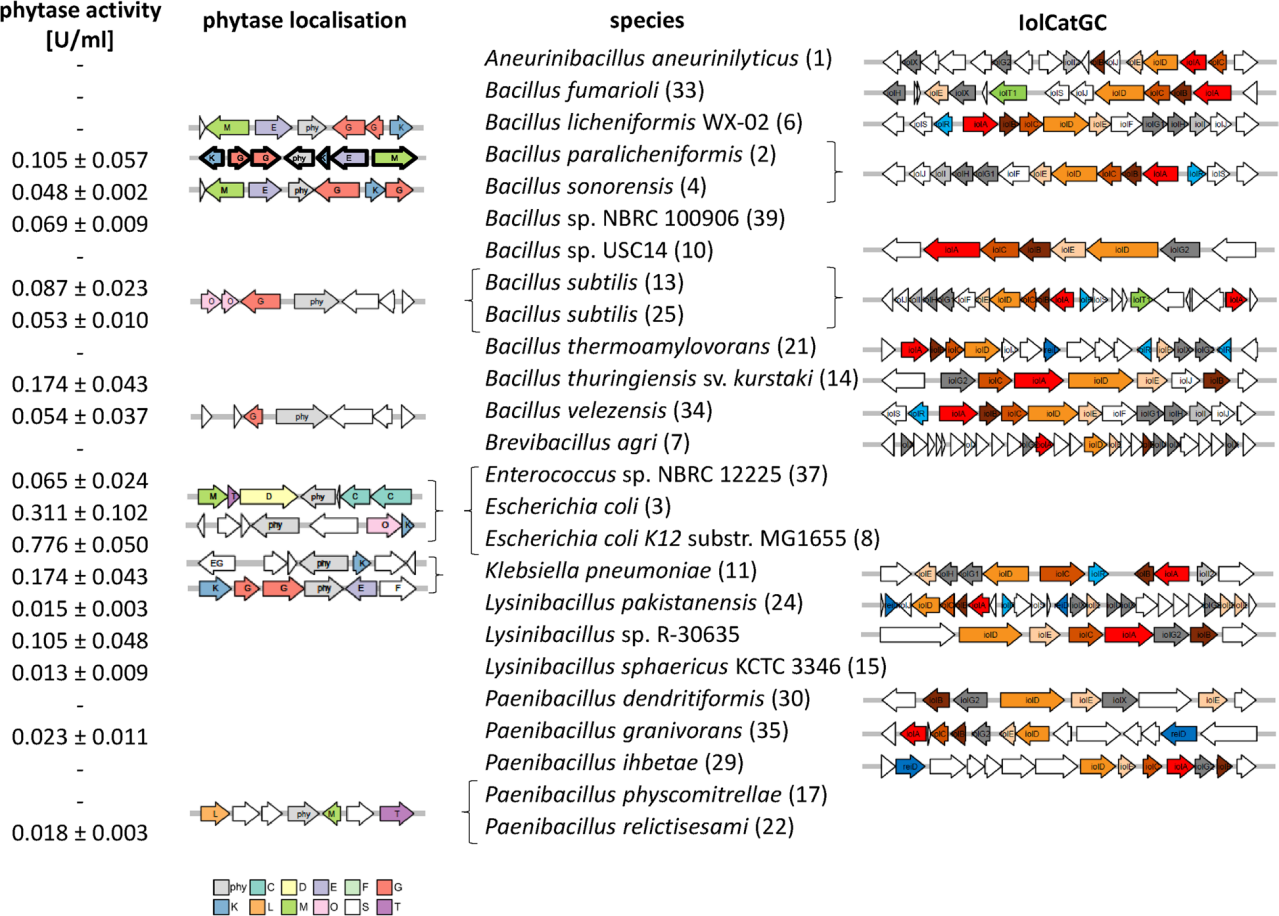
Geno- and phenotypes of characterized strains. Strains possessing a phytase gene and/or IolCatGC were selected from Table S3 for overview. *K. pneumoniae*, *Enterococcus* sp. and two *E. coli* strains each carry two phytase genes. Shown are phytase activities and the genetic neighborhoods of phytase genes, as determined by WGS. Numbers in brackets correspond to entries in Table S3, which provides detailed information on strain identification, growth conditions, and the presence of phytase genes and an IolCatGC. Genes are categorized by function as follows: C, energy production and conversion; D, cell cycle control, cell division, chromosome partitioning; E, amino acid transport and metabolism; F, nucleotide transport and metabolism; G, carbohydrate transport and metabolism; K, transcription; L, replication, recombination and repair; M, cell wall/membrane/envelope biogenesis; O, posttranslational modification, protein turnover, chaperones; phy, phytase; S, function unknown; T, signal transduction mechanisms. All strains grew with *myo*-Ins, but growth was weak in strains lacking IolCatGC. Gene names within IolCatGC are indicated

To identify genetic determinants involved in phytate metabolism, whole genome sequencing (WGS) was performed for all 40 isolates, and the resulting sequences were screened for the presence of *myo*-Ins catabolic gene clusters (IolCatGCs) [23] and phytase genes. The findings are presented in Table S3, including phytase accession numbers, and in Fig. 3. Unexpectedly, IolCatGCs responsible for *myo*-Ins degradation were identified in only 19 genomes, despite all 40 isolates growing on agar plates containing *myo*-Ins as the sole carbon and energy source. This discrepancy between phenotype and genotype suggests the existence of an alternative, yet unidentified, degradation pathway for this polyol [49], particularly in *B. subtilis*, *E. coli*, *Paenibacillus* spp., and *Lysinibacillus* spp. (Fig. 3). This hypothesis is supported by the observation that isolates from MM/*myo*-Ins plates lacking IolCatGCs displayed slower growth on *myo*-Ins and formed smaller colonies compared with those carrying an IolCatGC in their genome.

### Phytase activities of swine gut species

**Fig. 4:**
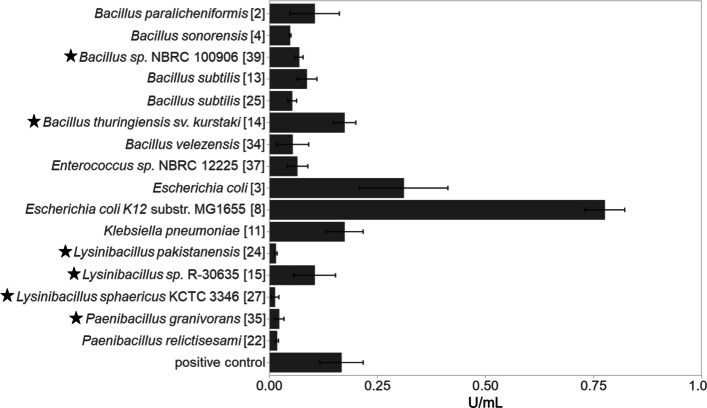
Quantification of phytase activities. Intracellular phytase activity was measured in crude protein extracts from bacteria isolated from piglets, following the method of Greiner et al. [16]. Numbers in brackets correspond to Table S3. Strains were grown individually in LB medium to stationary phase. *E. coli* DH5α served as a positive control. Asterisks indicate strains without a known phytase gene in their genome sequences. Data represent three biologically independent experiments; error bars show standard deviations

All 40 non-redundant isolates identified as *myo*-Ins degraders were examined for their ability to hydrolyse phytase. Isolates were grown under the isolation conditions and media specified in Table S3 until reaching the stationary phase, at which point maximal phytase activity was observed [50]. Using a modified assay to measure intracellular and periplasmic phytases [16], the highest phytase activity was detected with the protein extract of *E.* *coli* K12 substr. MG1655 (0.776 ± 0.005 U/ml) and *E. coli* (0.311 ± 0.102 U/ml), followed by *Bacillus thuringiensis* serovar kurstaki (0.174 ± 0.026 U/ml), *Klebsiella* *pneumoniae* (0.174 ± 0.043 U/ml), *B. paralicheniformis* (0.105 ± 0.057U/ml), and *Lysinibacillus* sp. R-30635 (0.105 ± 0.048 U/ml) (Fig. 4). The positive control *E. coli* DH5αA showed a high activity of 0.167 ± 0.050 U/ml, likely due to the presence of its AppA phytase (WP_001300464.1), which was described for the first time in *E. coli* [16]. *K. pneumoniae* possesses an AppA-like phytase, PhyK, which is assumed to completely dephosphorylate phytate [51, 52]. Quantitative phytase activity data are presented in Table S3 and in Fig. 3. WGS of the 40 isolates revealed that *K. pneumoniae*, *Enterococcus* sp., and two *E. coli* strains carry two phytase genes encoding a HAPhy, while nine strains harbour a single BPPhy. Twenty-seven strains lacked phytases with homology to any known phytase sequences. However, positive phytase test results suggest that at least six strains may produce a yet unidentified phytase or phosphatase (Table S3). *B. licheniformis* WX-02 and *Paenibacillus physcomitrellae* encode a phytase but did not exhibit detectable dephosphorylation activity, possibly due to unsuitable growth conditions or the presence of an extracellular phytase (see below). No conserved gene neighbourhood surrounding the phytase genes was observed across genera, and many phytase genes appear to be transcribed monocistronically (Fig. 3).

While most bacteria exhibit only intracellular phytase activity, extracellular phytases are frequently produced by *Bacillus* species, such as the 3-phytase of *B. subtilis* [53]. In this study, extracellular phytase activity was not quantified using our primary assay because protein precipitation via acetone interfered with the measurements. However, employing a commercial phytase detection kit, we detected extracellular phytase activity in *Bacillus pumilus, Brevibacillus borstelensis, and B. parabrevis*, none of which contained an identifiable phytase gene in their genome sequence. Taken together, the distinct phytase activities observed in this study suggest the presence of yet uncharacterized phytate-specific phosphatases, including enzymes lacking significant amino acid sequence homology to known phytases.

### Cross-feeding by phytases

The observation that seven of the 18 phytate degraders lack an IolCatGC, while 22 *myo*-Ins degraders show no phytate activity, suggests potential cross-feeding among gut bacteria. We therefore tested the hypothesis that phytase-positive (phytase^+^) commensals act synergistically in phytate dephosphorylation, releasing *myo*-Ins or its derivatives as potential growth substrates for IolCatGC-positive (IolCatGC^+^) bacteria in the swine gut. A consortium composed of seven strains—*E. coli, Bacillus sonorensis*, *Bacillus* *velezensis*, *B. licheniformis*, *K. pneumoniae, B. subtilis*, and *Enterococcus *sp. (Table S3)—identified as phytase^+^ by WGS and/or phytase activity assays, was co-cultivated in flasks in MM containing reduced phosphate levels (25% of standard concentration) to induce phytase production [54, 55]. Cultures were supplemented with 27.8 mM glucose and either with or without 10 mM sodium phytate. The supernatant was sterile-filtered and diluted 1:4 in M9 supplemented with 2 mM MgSO_4_ and 0.1 mM CaCl_2_ to produce a spent medium lacking an available carbon and energy source.

**Fig. 5:**
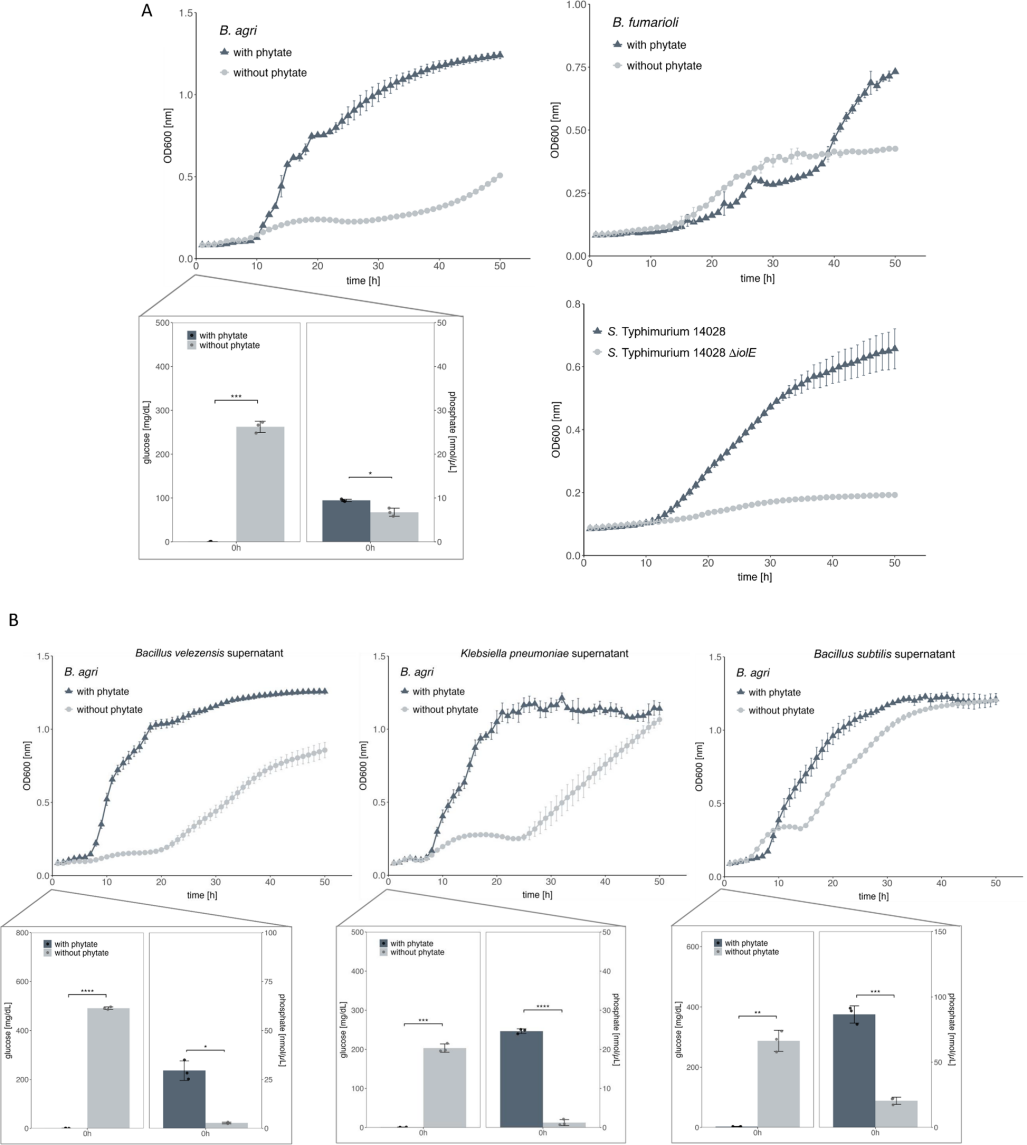
Cross-feeding. **A** The supernatant from a consortium of seven co-cultivated strains carrying phytase genes was sterile-filtered and diluted 1:4 in M9 supplemented with 2 mM MgSO_4_ and 0.1 mM CaCl_2_, with or without 10 mM phytate. Indicator strains *B. agri* and *B.* *fumarioli*, and *S. *Typhimurium were grown individually overnight in LB medium, then diluted 1:1000 into M9 supplemented with 25% consortium supernatant. Three biologically independent experiments were performed, error bars indicate standard deviations. *S*. Typhimurium strains 14,028 and 14,028 Δ*iolE*, the latter one unable to degrade *myo*-Ins, were cultivated similarly using supernatant from a consortium grown with phytate. Glucose and phosphate concentrations in the supernatant of the seven-strain consortium at time zero of indicator strain cultivation are shown as a breakout. Standard deviations of three independent measurements are shown. **B** Supernatants from *B.* *velezensis*, *K. pneumoniae*, and *B. subtilis* were tested for their ability to support the growth of indicator strain *B. agri*, as described in **A**. Glucose and phosphate concentrations in single-strain supernatants are indicated. *p*-values were determined Student’s *t*-test comparing supernatants with and without phytate. Asterisks indicate significant differences (*p < 0.05; **p < 0.01; ***p < 0.001; ****p < 0.0001)

Two indicator strains from the non-redundant list (Table S3), *Brevibacillus agri* and *Bacillus fumarioli*, were incubated in spent medium for 50 h at 37 °C. *B. agri*, identified as IolCatGC^+^ but phytase-negative (phytase^−^), grew well with spent medium derived from a consortium cultivated in the presence of phytate (Fig. 5A). In contrast, *B. agri* exhibited retarded growth and reached a lower OD_600_ when grown in spent medium from the consortium cultivated without phytate. The resulting diauxic-like growth curve suggested a metabolic shift toward the utilization of unidentified residual metabolites. A similar growth pattern was observed for *B. fumarioli* (phytase^−^, IolCatGC^+^) when exposed to consortional supernatant, although this strain displayed a prolonged lag phase when presumably utilizing *myo*-Ins. As further controls, both indicator strains were cultivated in MM with *myo*-Ins or phytate as the sole carbon and energy source (Fig. S1).

To further test synergistic cross-feeding toward an IolCatGC^+^, phytase^−^ pathogen, *Salmonella enterica* serovar Typhimurium strain 14,028 (*S.* Typhimurium 14,028) and its deletion mutant 14028Δ*iolE* that is unable to utilize *myo*-Ins [56] were grown in spent medium for 72 h at 37 °C. *S.* Typhimurium 14,028 reached an OD_600_ of 1.2 in spent medium, whereas the deletion of *iolE*, which is essential for *myo*-Ins degradation, resulted in severe growth attenuation (Fig. 5A).

To rule out the possibility that the control strains were using residual glucose and to confirm phytase activity, we measured glucose and free phosphate concentrations in the supernatant of the co-culture. Compared with cultures grown without phytate, the supernatant of cultures grown with phytate showed a marked decrease in glucose concentration from ~260 mg/dL to only 0.07–0.66 mg/dL and a slight, but significant, increase in phosphate concentration from ~7 nmol nm/µL to ~9 nmol/µL (Fig. 5A) (Table S4). We conclude that the consortium of seven phytase-producing strains has nearly depleted the available glucose and released free phosphate probably as a result of phytate dephosphorylation.

To determine whether phytate dephosphorylation to *myo*-Ins or a related derivative can be attributed to individual strains, each member of the consortium was tested for its ability to cross-feed *B. agri*. Supernatants from seven cultures, grown in flasks with and without phytate, were used as growth media for *B. agri*. The strongest growth-promoting effects on the test strain were observed with the supernatants from *K. pneumoniae*, which was previously suggested to release *myo*-Ins from phytate [52], and from *B.* *velezensis* (Fig. 5B). In contrast, supernatants from *B. subtilis* and the remaining four strains produced only a slight increase in the logarithmic growth phase of *B. agri*. Analysis of the supernatants revealed strong decreases in glucose concentrations and significant increases in phosphate concentrations (Fig. 5B) (Table S4). The higher glucose levels in the supernatant of *B.* *velezensis* and *B. subtilis*, compared with those of the consortium, are likely due to growth inhibition of these strains under low-phosphate conditions such as in the absence of phytate. To finally demonstrate that the supernatant from cultures grown not only in flasks but also under anaerobic conditions, the consortium as well as *K. pneumoniae* were grown in an anoxic chamber. Feeding the supernatants to *B.* *agri* and *B.* *fumarioli* resulted in growth curves similar to those described above (Fig. S2).

Taken together, these findings demonstrate that consortium members, particularly *B.* *velezensis* and *K. pneumoniae,* dephosphorylate phytate, thereby releasing *myo-*Ins that can be utilized by IolCatGC^+^ *B.* *agri*, *B.* *fumarioli*, and *S.* Typhimurium. This suggests multiple cross-feeding activities in the gut that contribute to phytate metabolism.

## Discussion

Culture-independent sequencing methods have generated extensive gene catalogues and advanced our understanding of the microbiome’s functional diversity of the pig microbiome [57, 58]. The pig gut microbiota is dominated by the phyla Bacillota and Bacteroidota, with the genera *Prevotella*, *Bacteroides*, and *Ruminococcus* being particularly abundant and playing important functional roles [57]. For instance, the succinate producer *Prevotella copri* has been shown to improve blood sugar regulation in mice [59], and *B. subtilis* has been shown to exert probiotic effects in pigs and promote host health [60]. Numerous other commensal bacteria also contribute to maintaining intestinal function [57]. However, limited gene annotation and unclear gene functions necessitate the use of culturomics and subsequent functional studies [45–47]. Wang et al. reported that *Bacillus* was primarily detected through aerobic cultivation [47]. Culture-independent 16S sRNA analysis is less effective for isolating *Bacillus*, particularly in spore form, which may lead to the omission of core microbiota members. Our feces analyses confirm that culture-dependent isolation of microbiota members does not fully reflect the community composition revealed by 16S rRNA-based, culture-independent analysis [61]. Within the dominant phylum Bacillota, *Bacillus* was most abundant in our study, followed by *Aneurinibacillus, Lysinibacillus*, and *Paenibacillus*. Large-scale analyses indicated that 40% of *Bacillus* genomes have the genetic capability to degrade *myo*-Ins [23].

Microorganisms utilizing inositol phosphates are known to be ubiquitous in both terrestrial and aquatic environments [62]. However, our understanding of phytate-dephosphorylating microorganisms remains limited, largely due to the scarcity of metabolic studies and the difficulty of isolating candidate strains from diverse ecological habitats. In this study, we focused on the swine intestine as an underexplored environment for phytate- and *myo*-Ins-utilizing bacteria. Under aerobic and anaerobic conditions, 33% to 70% and 17% to 39% of all cells grown on LB, respectively, were found to utilize *myo*-Ins. Using a large-scale bioinformatic approach, we recently estimated that approximately 10.3% of bacterial species in the swine gut possess the capacity to degrade *myo*-Ins [23]. These findings suggest an enrichment of species, as well as their cell numbers, in the piglet gut that are able to grow on *myo*-Ins. This hypothesis is in line with the observation that certain taxa exhibited a higher abundance on *myo*-Ins medium than others, although potential biases in strain abundance due to the use of rich medium cannot be excluded. Further limitations of our culturomics approach were the small animal cohort, a lack of community-level metabolomics, and a preference for aerobic versus anaerobic conditions. Nevertheless, a positive correlation between the culturability and relative abundance as determined by 16S rRNA analysis has been reported [63], and two media have been shown to be sufficient for isolating the majority of gut bacteria [64]. Most of the bacteria listed in Table S3 were isolated from plates incubated aerobically, primarily because anaerobic strains grew poorly when *myo*-Ins was provided as the sole carbon and energy source. The use of a selective MM with *myo*-Ins represents an example of metagenome-guided culturomics [65]. Further studies involving distinct dietary groups will be needed to determine whether a phytate-rich diet promotes a shift in the microbiota toward bacteria capable of utilizing *myo*-Ins.

Pigs are widely used as model organisms, yet the functional interactions within their intestinal microbiome remain poorly understood. Recent studies have identified links between the gut microbiota and feed efficiency in pigs [66, 67]. To shed further light on metabolic synergisms, we searched for phytase producers in piglet fecal samples. Anaerobic environments have been predicted to be important sources of novel phytate-degrading microorganisms [68]. The distinct phytase activities observed among 40 strains isolated from piglet feces, along with discrepancies between genotype and phenotype, such as the absence of identifiable phytase genes in strains capable of dephosphorylating phytate, strongly suggest the presence of yet unknown phytases and phytate-specific phosphatases. Further characterization of these and other strains will aid in the discovery of novel, and potentially unexpected, phytase functions, as well as in the optimization of phytase applications. Phytases hold substantial potential in animal and human health and nutrition, for example, by improving iron and phosphate availability and enabling the dephytinization of infant formula, although possible drawbacks of phytase implementation, such as the loss of antioxidant function, should be considered. Industrial applications including food processing, biofuel production, soy milk dephytinization, and paper production are less controversial [8]. As a future perspective, phytases may also contribute to reducing eutrophication by enabling phosphate recovery from (agricultural) wastewater.

We propose that bacterial dephosphorylation and subsequent *myo*-Ins utilization play a hitherto underestimated role in gut microbiota-host interactions. Reported physiological concentrations of *myo*-Ins are either absent from the literature or very low, for example, in plasma (~20–40 µM) [69]. Free *myo*-Ins is rapidly absorbed by the host [70] and likely also by the microbiota. In contrast, dietary phytate intake in adults ranges from approximately 0.65 to 2.6 g per day and can reach up to 7.5% of body weight in livestock feed. This substantial phytate input supports the assumption that *myo*-Ins represents a significant growth substrate for gut commensals. To access potentially the carbon and energy contained in its *myo*-Ins component, bacteria require a phytate-degrading enzyme. According to our data, a substantial proportion of gut commensal bacteria are phytase+, but IolCatGC^−^, and vice versa, tempting us to speculate that extensive cross-feeding occurs in the swine gut. This hypothesis rests on the assumption that limitations in carbon, phosphorus, and energy sources as encountered in the gut confer a significant competitive advantage to commensal microorganisms capable of degrading phytate and/or *myo*-Ins. Indeed, *Mitsuokella jalaludinii* has been identified as an efficient phytate degrader that provides *Anaerostipes rhamnosivorans* with *myo*-Ins [20]. In the present study, we demonstrate that additional commensals also interact through the metabolic conversion of phytate, and we hypothesize that an even huger number of gut bacteria may contribute to or benefit from this cross-feeding activity. This group may comprise those pathogenic enterobacteria in whose genomes IolCatGCs were identified [23]. These findings are consistent with the predictions of optimal foraging theory, which states that an efficient microbial community should adjust its foraging strategy for different nutrients to maximize the acquisition of limiting nutrients while avoiding excessive acquisition of non-limiting nutrients [1].

## Conclusion

The results of this study provide new insights into the ecological significance of phytase^+^ microorganisms within the intestinal ecosystem. The diversity of phytase activities detected among gut microbes suggests that additional bacterial species and phytases from diverse environments remain to be characterized, with potential applications in animal nutrition. Our experiments demonstrate that *myo*-Ins serves as a common carbon and energy source for many gut bacteria, and that phytate degraders such as *K. pneumoniae* and *B.* *velezensis* can fuel the metabolism of *B.* *agri*, *B.* *fumarioli*, and likely many other commensals. These findings shed light on a previously overlooked form of multiple cross-feeding within microbial communities. The presence of large groups of phytate and/or *myo*-Ins degrading bacteria is thought to contribute to the selective degradation of an important dietary component, thereby helping to balance the elemental requirements of both commensal microbes and their hosts.

## Materials and methods

### Culturomics

**Fig. 6:**
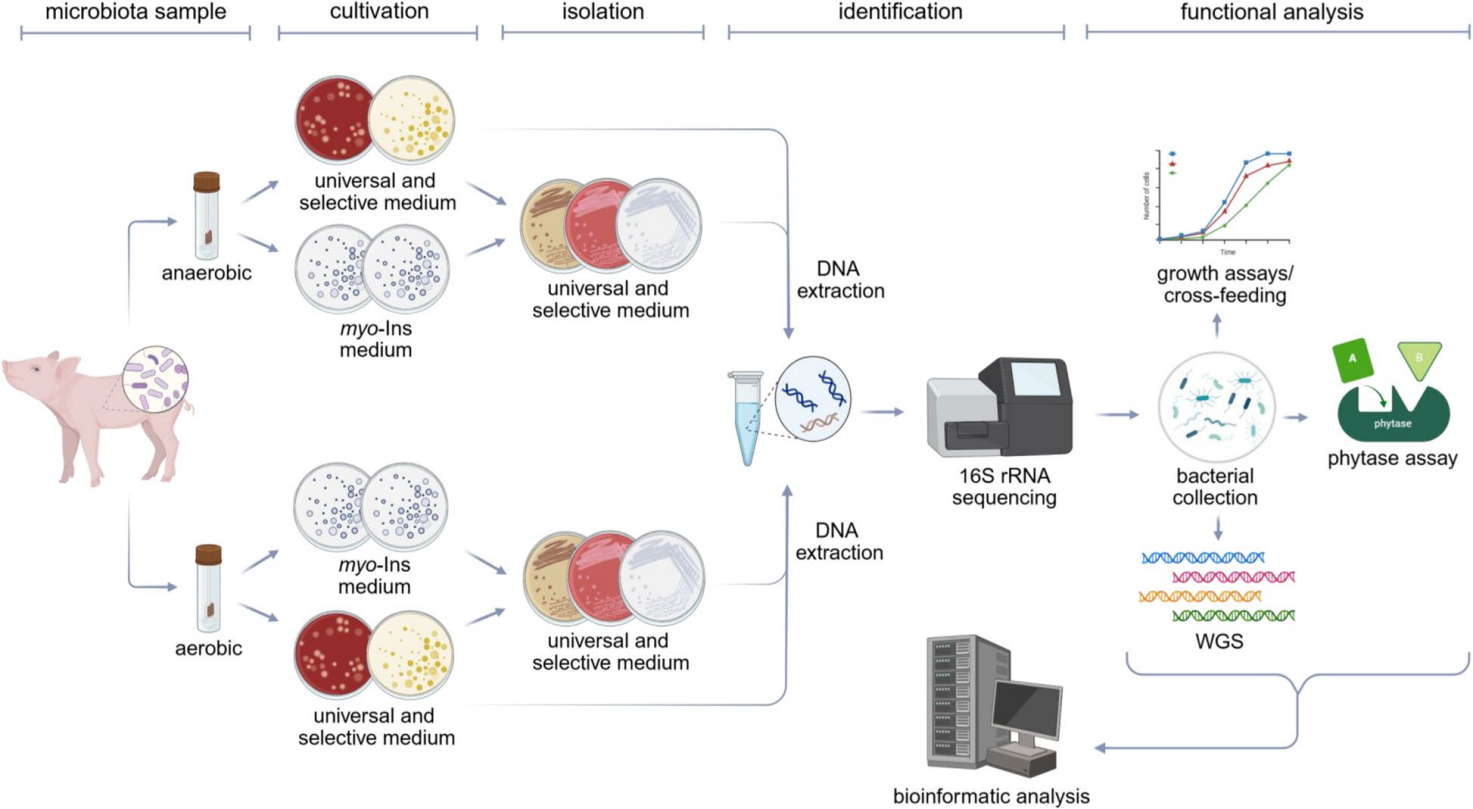
Culturomics approach. The figure summarizes the techniques applied here to isolate and characterize gut isolates from fecal samples of piglets. Created in BioRender (2025; https://BioRender.com/8zfpdjt)

An overview of the culturomics approach applied in this study is shown in Fig. 6.

### Feces samples used in this study

For gut strain isolation, fresh fecal samples were collected directly from the rectum of three piglets belonging to a control group of a feeding experiment [40] and stored at −20 °C. In addition, intestinal contents from the ileum, caecum, and colon were collected during the section. The pig study protocol was approved by the Thüringer Landesamt für Verbraucherschutz, Bad Langensalza, Germany (approval number 22–2684-04-BFI-17-001).

### Culture media for strain isolation

Details of media are listed in Table S2; if appropriate, media were supplemented with 15 g agar. Media used were: Brain heart infusion (BHI; Carl Roth GmbH, Karlsruhe, Germany; Oxoid, Wesel, Germany); Colombia agar with sheep blood (ColSB; Oxoid); MacConkey agar with salt (MAC; Oxoid); Wilkens Chalgren anaerobe agar with neomycin (WCA; Oxoid); lysogeny broth (LB; 10 g/L tryptone, 5 g/L yeast extract and 5 g/L NaCl); M9, salt medium 10× stock (128 g/L Na_2_HPO_4_ × 7H_2_O, 30 g/L KH_2_PO_4_, 5 g/L NaCl, 10 g/L NH_4_Cl); minimal medium (MM; M9 medium supplemented with 2 mM MgSO_4_, 0.1 mM CaCl_2_, 55.5 mM (1% [wt/vol]) *myo*-Ins, or 27.8 mM (0.5% [wt/vol]) glucose); Schaedler anaerobe agar with sheep blood, hemin and vitamin K (SCH; Thermo Fisher Scientific, Darmstadt, Germany); de Man-Rogosa-Sharpe agar (MRS; Oxoid); fastidious anaerobe agar with horse blood (F.A.A.; Thermo Fisher Scientific); anaerobe recovery and isolation agar with 5% horse blood and neomycin (A.R.I.A.; Thermo Fisher Scientific).

### Bacterial isolation, cultivation, and storage

For aerobic strain isolation, 0.1 g of fecal sample was homogenized in either BHI, M9, *myo*-Ins, or LB, and enriched by overnight growth. Reaction tubes were left to stand for 1 min to allow debris sedimentation. The supernatant was used to prepare ten-fold serial dilutions in sterile PBS, and 100 µL aliquots were plated onto various solid media (see above). Plates were incubated aerobically at 37 °C for at least one day and up to 8 days before colony selection. Pure colonies were obtained by re-streaking single colonies at least three times prior to assessing colony morphology. All isolates were finally re-streaked on *myo*-Ins plates.

For anaerobic isolation, culture media were pre-incubated for at least 48 h in an anaerobic workstation (Don Whitley Scientific, Bingley, UK) under an atmosphere of N_2_ (89.3%), CO_2_ (6%), and H_2_ (4.7%). Sample processing and culturing were performed entirely under anaerobic conditions. For long-term storage, cryo-stocks were prepared by suspending freshly grown colonies in culture medium mixed 1:1 with sterile medium containing 20% (v/v) glycerol.

### Standard techniques

DNA manipulations and isolation of chromosomal DNA were performed according to standard protocols [71].

### Bacterial species identification

Bacterial species were identified by amplifying the 16S rRNA gene amplicon using primer 27f and 1492r [43]. Amplicons were subjected to Sanger sequencing (Eurofins, Ebersberg, Germany) with primer 27f following PCR product purification (Nucleo Spin Clean Up, Machery-Nagel, Düren, Germany). The closest related species with validly published names were determined using EzBioCloud [72], NCBI Blast, and the SILVA aligner [73]. Strain identification by SILVA was further corroborated using MetaPhlan v4. A 16S rRNA gene sequence identity threshold of 95.0% in EzBioCloud was applied as a genus-level cut-off [74].

### Preparation and use of spent medium

To produce a spent medium containing available carbon and energy sources, fecal strain consortia and single strains were grown aerobically or anaerobically in flasks to stationary phase in 20 mL MM with 25% of the standard phosphate concentration, with or without supplementation of 10 mM sodium phytate (Sigma-Aldrich, Schnelldorf, Germany). Bacterial supernatants were obtained via centrifugation (11,000 × g/20 min/RT) and sterile filtered through a 0.2 µm filter. To assess the synergy between phytase-producing gut isolates and *myo*-Ins-catabolizing bacteria, cultures were incubated at 37 °C in spent media consisting of M9 medium supplemented with 2 mM MgSO_4_, 0.1 mM CaCl_2_, and 25% [v/v] bacterial supernatant for at least 72 h in an automated plate reader (Epoch2T; BioTek, Bad Friedrichshall, Germany). Glucose and phosphate concentrations were quantified using assay kits (Invitrogen, Sigma-Aldrich). Limitations of the glucose kit required dilution at higher glucose concentrations, resulting in measurement errors that were re-calculated. Figures were created with the R package ggplot2 (v.3.5.1).

### Profiling of plate microbiota

Pig fecal samples were homogenized in LB medium and enriched by overnight growth under aerobic and anaerobic conditions. Debris was sedimented for 1 min at 1 × g, and the supernatant was used to prepare ten-fold serial dilutions in sterile PBS, which were plated onto LB and MM/*myo*-Ins solid media. Plates were incubated aerobically or anaerobically at 37 °C, and colony-forming units were enumerated. Plate surfaces were washed with 2 mL PBS, and the wash solution was transferred into reaction tubes. After centrifugation at 11,000 × g for 5 min at room temperature, cell pellets were resuspended in lysis buffer (Nucleo Spin Tissue, Machery-Nagel), and genomic DNA was isolated. Sequencing of 16S rRNA gene amplicons was performed at the ZIEL Core Facility: Microbiome at TU Munich [75]. Raw reads were processed using the Integrated Microbial Next Generation Sequencing (IMNGS) pipeline [76] based on UPARSE [77]. Sequences were demultiplexed, trimmed to remove bases with a quality score < 10, and paired. Assemblies < 350 nucleotides (nt) and >500 nt, or with an expected error > 2, were excluded. Remaining reads were trimmed by 5 nucleotides at both ends to avoid regions with distorted base composition. Pairing, filtering, and operational taxonomic unit (OTU) clustering at 97% sequence identity were performed with USEARCH 11.0 [78]. OTUs with a relative abundance ≥ 0.25% in at least one sample were retained. Non-16S sequences were removed using SortMeRNA (v4.2) [79]. Taxonomy assignments were made at a 70% confidence level, integrating results from SINA (v1.6.1) [73]. Downstream analyses were performed in R using the Rhea pipeline [80]. Microbiota control data were identical to those reported in Bublitz et al. [40]. Stacked bar charts were generated with the R package ggplot2 (v.3.5.1).

### Whole-genome sequencing and phytase gene identification

Illumina sequencing data of chromosomal DNA were analysed using the WGSBAC pipeline [https://gitlab.com/FLI_Bioinfo/WGSBAC, (accessed 1 February 2024)]. Quality control was performed with FastQC (v. 0.11.7). De novo assemblies were generated using the Shovill pipeline v. 1.1 [81] and evaluated with QUAST (v. 5.0.2) [82]. Gene annotation for all assemblies was performed with Bakta (v. 1.9.4) [83]. Gene maps depicting phytase gene regions and IolCatGCs were constructed with the R package gggenes (v. 0.4.1), with IolCatGC information obtained from [23].

To identify phytase genes, genome-wide annotation was conducted using Bakta with its full reference database. Additionally, BLASTp searches were performed for all predicted protein-coding sequences against reference phytase sequences from NCBI: AIE90144.1 and NP_415500.1 (classified as HAPhy) and AFQ59979.1 (classified as BPPhy). Sequences showing ≥30% percentage identity and ≥50% query coverage were assigned to the corresponding phytase class (HAPhy, BPPhy) based on the best hit.

### Protein extraction

Isolates were grown in 20 mL of the medium indicated in Table S3 at 37 °C until reaching stationary phase, under either aerobic or anaerobic conditions, depending on the isolation requirements. Bacterial cells and supernatants were separated by centrifugation at 11,000 × g for 10 min at4 °C. The resulting cell pellets were resuspended in 150 µL of lysozyme treatment buffer [2 mM EDTA, 1.2% Triton X-100, 200 mM Tris–HCl (pH 8), 20 mg/ml lysozyme], then ultrasonicated three times for 1 min at 37 kHz (with degassing). Cell debris was removed by centrifugation at 11,000 × g for 10 min at 4 °C. The crude protein extracts were stored at −20 °C in 100 mM sodium acetate buffer (pH 5.0).

### Phytase assay

Phytase activity was determined as described by Greiner et al*.* [84]. Briefly, 50 µL of protein extract was incubated with 350 µL of sodium phytate solution (Sigma Aldrich; purity > 79%, total phosphorus content 19%–25%) in 100 mM sodium acetate buffer, pH 5.0 for 60 min at 37 °C. As a negative control, 50 µL of crude protein extract was incubated in sodium acetate buffer (100 mM, pH 5.0) without sodium phytate as the negative control. The reaction was terminated by adding 1500 μL of a freshly prepared stop solution containing acetone, sulfuric acid, ammonium molybdate (10 mM) (2:1:1), and 100 μL of citric acid (1 M). After centrifugation at 10,000 × g for 10 min, the absorbance of the supernatant was measured at 355 nm with a UV/Vis spectrophotometer. Blanks were prepared by adding the stop solution prior to substrate addition. Protein concentrations were determined using the Bradford assay with bovine serum albumin as the standard [85]. One unit of phytase activity was defined as the release of 1 μmol of phosphorus per minute. Additionally, phytase activity was assessed using a commercial phytase kit (Neogen, Moers, Germany).

## Supplementary Information

Supplementary Material 1. Figure S1: Growth controls with *myo*-Ins and phytate.

Supplementary Material 2. Figure S2: Anaerobic cultivation for supernatant yield.

Supplementary Material 3. Table S1: Colony forming units.

Supplementary Material 4. Table S2: List of media.

Supplementary Material 5. Table S3: Non-redundant isolates_extended.

Supplementary Material 6. Table S4: Data of Fig. 5.

## Data Availability

The sequencing data of the study are accessible under the ENA Bioproject PRJEB89380.
