# Supplementary figures and images for: A culturomics approach reveals cross-feeding capacity of intestinal pig bacteria upon release of inositol from phytate
Source: Microbiome. 2026 Jan 21;14:44. doi: 10.1186/s40168-025-02313-5 (PMC12838507; doi:10.1186/s40168-025-02313-5)

## Slide 1
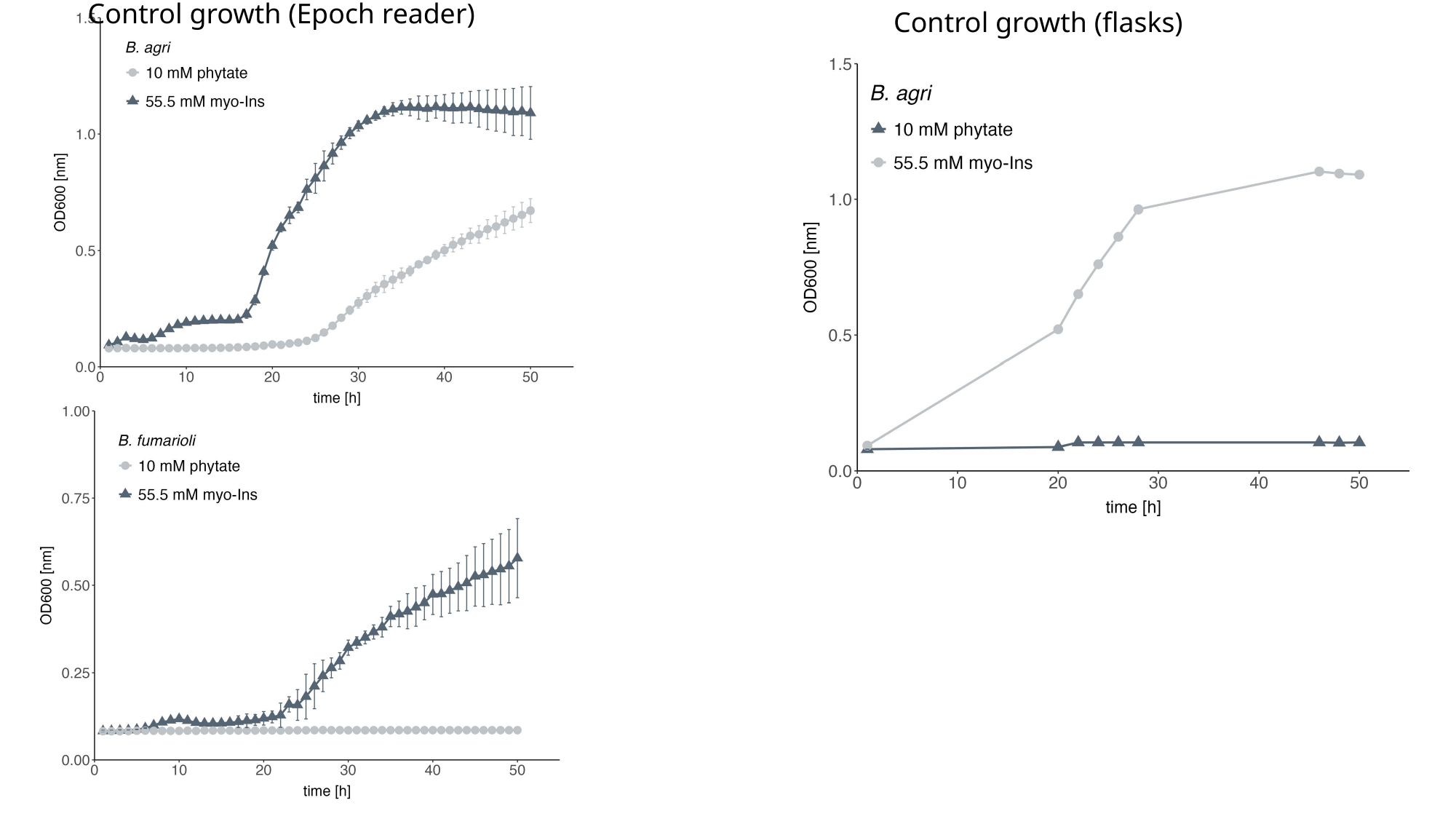

Control growth (flasks)
Control growth (Epoch reader)

Supplement: Supplementary file 2 — Supplementary Material 1. Figure S1: Growth controls with myo-Ins and phytate. [file 40168_2025_2313_MOESM1_ESM.pptx]
