# Supplementary material for: A culturomics approach reveals cross-feeding capacity of intestinal pig bacteria upon release of inositol from phytate
Source: Microbiome. 2026 Jan 21;14:44. doi: 10.1186/s40168-025-02313-5 (PMC12838507; doi:10.1186/s40168-025-02313-5)
Supplement: Supplementary file 3 — Supplementary Material 2. Figure S2: Anaerobic cultivation for supernatant yield. [file 40168_2025_2313_MOESM2_ESM.pptx]

## Slide 1
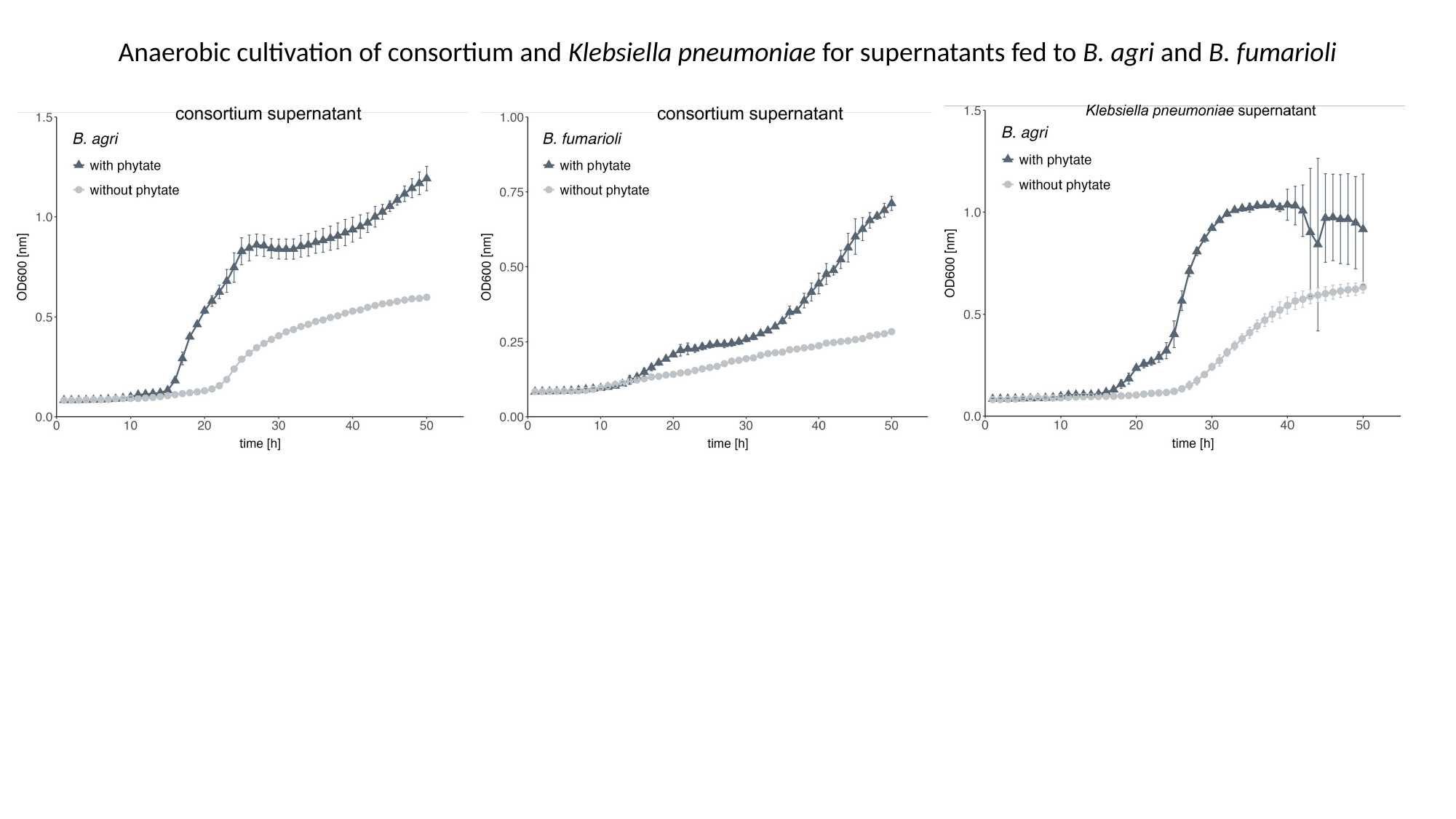

Anaerobic cultivation of consortium and Klebsiella pneumoniae for supernatants fed to B. agri and B. fumarioli
